# Inhibiting the NLRP3 Inflammasome With MCC950 Ameliorates Isoflurane-Induced Pyroptosis and Cognitive Impairment in Aged Mice

**DOI:** 10.3389/fncel.2018.00426

**Published:** 2018-11-19

**Authors:** Yunxia Fan, Liwu Du, Qun Fu, Zhiqiang Zhou, Jingyu Zhang, Guomin Li, Jing Wu

**Affiliations:** ^1^Department of Anesthesiology, Jintan Hospital, Jiangsu University, Changzhou, China; ^2^Department of Anesthesiology, Nanjing Branch of Shanghai Changzheng Hospital, The Second Military Medical University, Nanjing, China; ^3^Department of Anesthesiology, Jinling Clinical Medical College of Nanjing Medical University, Nanjing, China; ^4^Jiangsu Key Laboratory of Molecular Medicine, Medical School of Nanjing University, Nanjing, China

**Keywords:** NLRP3, neuroinflammation, pyroptosis, cognitive impairment, general anesthesia, aging

## Abstract

Nod-like receptor protein 3 (NLRP3) inflammasome activation has been implicated in the pathogenesis of general anesthesia (GA)-induced neuroinflammation and cognitive impairment in aged rodents. However, the cellular basis for cognitive impairment is still not fully understood, and effective pharmacologic agents targeting the NLRP3 inflammasome during GA are lacking. This study explores the protective effects of the NLRP3 inflammasome inhibitor MCC950 on pyroptosis and cognitive impairment in aged mice exposed to isoflurane. Seventy-two 15-month-old male C57BL/6 mice were randomized to receive 2 h of 1.5% isoflurane plus 30% oxygen (O_2_) or 30% O_2_ alone, respectively. MCC950 (10 mg/kg) or vehicle was intraperitoneally administered 30 min before gas inhalation. Brain tissues were harvested for histochemical analysis and biochemical assays. Learning and memory abilities were evaluated by behavioral tests. We found that isoflurane GA caused upregulations of hippocampal NLRP3, cleaved caspase-1, interleukin-1β (IL-1β), and IL-18 and the activation of pyroptosis, which is NLRP3 inflammasome-dependent; this consequently gave rise to neuronal damage and cognitive impairment in aged mice. Interestingly, pretreatment with NLRP3 inflammasome inhibitor MCC950 not only provided a neuroprotective effect against the inflammasome activation but also ameliorated pyroptosis and cognitive impairment in aged mice exposed to isoflurane. Our data demonstrate that pyroptosis is involved in NLRP3 inflammasome-mediated isoflurane-induced cognitive impairment in aged mice and suggest that inhibiting the NLRP3 inflammasome with MCC950 may have clinically therapeutic benefits for elderly patients undertaking GA.

## Introduction

Postoperative cognitive dysfunction (POCD) is a common complication associated with significant morbidity and mortality in elderly patients (Berger et al., 2015; Moskowitz et al., 2017). General anesthesia (GA)-induced neuroinflammation is increasingly emphasized as a key factor in the pathogenesis of POCD (Hovens et al., 2014; Umholtz and Nader, 2017; Skvarc et al., 2018). The NOD-like receptor protein 3 (NLRP3) inflammasome, an intracellular multiprotein complex, is the best recognized and most widely studied inflammasome. It regulates the cleavage of caspase-1, which controls the processing and secretion of the pro-inflammatory cytokines IL-1β and IL-18 (He et al., 2016a; Dempsey et al., 2017; Jiang et al., 2018). These cytokines are crucial in initiating or amplifying the innate immune response and neuroinflammation following GA. We previously showed that isoflurane GA induced the activation of NLRP3, the cleavage of caspase-1 and the secretion of IL-1β and TNF-α in the hippocampus of aged mice (Wu et al., 2015a) and suggested that the NLRP3 inflammasome could be a promising molecular target for POCD treatment (Li et al., 2014; Wu et al., 2015a). MCC950 is a highly potent NLRP3 inhibitor and was first introduced as a specific anti-inflammatory compound in 2015 (Coll et al., 2015). The novel compound MCC950 has been clearly shown to be neuroprotective in multiple neurological disorders, including ischemic and degenerative diseases (Ye et al., 2017; Qi et al., 2018). However, the effects of MCC950 on GA-induced neuroinflammation and cognitive impairment are unknown.

Pyroptosis is a novel inflammatory form of programmed cell death. This type of cell death was recently characterized to be NLRP3 inflammasome-dependent and can be triggered in the canonical (caspase-1-mediated) and non-canonical (caspase-11-mediated) inflammasome signaling pathways (He et al., 2016a; Dempsey et al., 2017; Jiang et al., 2018). Accumulating evidence suggests that pyroptosis is involved in the pathogenesis of many infectious and non-infectious diseases (Shi et al., 2015; Aglietti and Dueber, 2017), and MCC950 can inhibit the NLRP3 inflammasome to further prevent pyroptosis (Mangan et al., 2018; Shen et al., 2018). However, little is known about the function of pyroptosis in GA-mediated neurotoxicity and cognitive deficits. Uncovering the mechanisms of this process may provide a novel avenue for POCD treatment. Thus, an effective intervention, MCC950, is emerging.

In the present study, we hypothesize that NLRP3 inflammasome-dependent pyroptosis is involved in the mechanism of isoflurane-induced cognitive impairment in aged mice and that inhibiting the NLRP3 inflammasome with MCC950 may ameliorate isoflurane-induced pyroptosis and cognitive impairment.

## Materials and Methods

### Animals

Seventy-two 15-month-old male C57BL/6 mice were purchased from Jiangsu University, Zhenjiang, China. All experimental procedures and protocols were reviewed and approved by the Animal Investigation Ethics Committee of Jiangsu University and were done in accordance with the Guidelines for the Care and Use of Laboratory Animals from the National Institutes of Health, United States. The mice were housed in a room maintained under stable environmental conditions with a temperature of 22–24°C and a 12-h light/dark cycle.

### Experimental Protocols

The mice were randomly assigned to one of the following four groups (*n* = 18 each): control, control + MCC950, isoflurane, and isoflurane + MCC950. MCC950 (10 mg/kg, China Peptides Co, Ltd, Shanghai, China) or phosphate-buffered saline (PBS, vehicle) was intraperitoneally administered to the mice in a volume of 0.4 mL/kg 30 min before gas inhalation. The dose of MCC950 was chosen based on previous optimization showing that the intraperitoneal administration of MCC950 at 10 mg/kg presented neuroprotective effects in mice models (Ren et al., 2018; Xu et al., 2018; Zhai et al., 2018). Anesthesia was induced by placing the mice in an anesthetizing chamber prefilled with 1.8% isoflurane plus 30% O_2_ for 10 min that was then changed to 1.5% isoflurane for 110 min. For control experiments, 30% O_2_ was delivered for 2 h at an identical flow rate (Wu et al., 2015a; Wu et al., 2016). The composition of the chamber gas was continuously monitored using a Datex^TM^ infrared analyzer (Capnomac, Helsinki, Finland). The mice were kept normothermic throughout the experiment.

Six mice in each group were decapitated 24 h after gas inhalation, and the brain was rapidly removed and separated into two halves for histochemical analysis and biochemical assays. The remaining 12 mice in each group were subjected to behavioral tests 24 h and 1 week after gas inhalation.

### Hematoxylin and Eosin (HE) Staining

Half of each mouse brain (*n* = 6 for each group) underwent HE staining and immunohistochemical analysis 24 h after gas inhalation. Brain tissues were immersed in 4% paraformaldehyde and embedded using paraffin. The tissues were sliced into 4 mm sections until use.

HE staining was performed as follows: hematoxylin staining for 5 min, 75% hydrochloric acid alcohol solution for 30 s decoloring, eosin staining for 5 min, and 90% ethanol for 35 s decoloring. Normal neurons have a relatively large cell body that is rich in cytoplasm with one or two large round nuclei, while damaged cells show shrunken cell bodies, pyknotic nuclei, dark cytoplasm, and many empty vesicles. Hippocampal neuronal damage was evaluated using a standard semi-quantitative scale (Tang et al., 2016). Briefly, grade 0 = no damage to any hippocampal subregion, grade 1 = scattered neurons are damaged in the CA1 (cornu ammonis) subregion, grade 2 = moderate numbers of neurons are damaged in the CA1 subregion (<50% neurons damaged), grade 3 = severe damage (>50% of cells affected) to pyramidal cells in the CA1 subregion, and grade 4 = extensive cell damage in all hippocampal regions. Four random high-power (at ×400 magnification) visual fields from each brain slice were checked. Evaluation of cells with nuclear pyknosis and morphologic abnormality was performed by two pathologists blind to the treatment groups.

### Immunohistochemistry

Paraffin sections were then deparaffinized and hydrated using the following steps: 10 min in xylene twice; 5, 10, 10, and 10 min in 100, 95, 85, and 70% ethanol, respectively; and 5 min in PBS at room temperature repeated three times. Antigen retrieval was achieved by boiling the sections in 10 mM sodium citrate for 10 min in a microwave oven. The sections were then washed with PBS three times and treated with 3% H_2_O_2_-methanol for 15 min. Immunostaining was performed by incubation with antibody against NLRP3 (1:100; Servicebio Technology Co., Ltd., Wuhan, China) for 2 h. The sections were then washed three times and incubated with secondary antibody labeled with horseradish peroxidase for 30 min at room temperature. Cells with brownish-yellow cytoplasm were counted as positive cells. The numbers of NLRP3 immunoreactive cells in the hippocampal CA1 region were counted and analyzed in four microscopic fields (at ×400 magnification) by an investigator blinded to the treatment conditions.

### TUNEL Fluorescent Assay

TUNEL assay was conducted with an *in situ* Cell Death Detection Kit (Roche Inc., Indianapolis, IN, United States) following the protocols. Sections were counterstained by Anti-NeuN Antibody (1:200, Merck Millipore, Hong Kong, China) for 3 min. The sections were then washed with PBS three times and covered by microscopic glass with Antifade Mounting Medium (Beyotime Institute of Biotechnology, Shanghai, China) for further study. The numbers of TUNEL-positive cells in the hippocampal CA1 region were counted and analyzed (at ×400 magnification) by an investigator blinded to the treatment conditions.

### Western Blotting Analysis

The hippocampus from the other half of the brain (*n* = 6 for each group) was isolated and homogenized for biochemical assays. Equal amounts of protein (35 μg/lane) were electrophoretically separated and blotted onto nitrocellulose membranes. Protein levels were determined via incubation against antibody of NLRP3 (1:600; Servicebio Technology Co., Ltd.), ASC (1:200; Santa Cruz, CA, United States), caspase-1 (1:500; Servicebio Technology Co., Ltd.), gasdermin-D (GSDMD, 1:500; Abcam, United Kingdom), IL-1β (1:200; Santa Cruz, United States), IL-18 (1:200; Santa Cruz, CA, United States), or β-actin (1:1,000, Cell Signaling Technology, United States). Bands were visualized by enhanced chemiluminescence and quantified with the Image Quant Software (Syngene).

### Enzyme-Linked Immunosorbent Assay

The quantifications of IL-1β and IL-18 in the hippocampus were performed using an enzyme-linked immunosorbent assay (ELISA) kit following the manufacturer’s instructions (Servicebio Technology Co., Ltd).

### Open Field Test

The open field test and fear conditioning test were conducted 24 h after gas inhalation in a sound-isolated room by a single investigator who was blinded to the group assignment. To evaluate the anxiety behavior and general locomotor activity, each mouse (*n* = 12 for each group) was gently placed in the center of a white plastic chamber (40 cm × 40 cm × 40 cm) for 5 min, and exploratory behavior was automatically recorded by a video tracking system (XR-XZ301, Shanghai Softmaze Information Technology Co., Ltd., Shanghai, China). After each test, the arena was cleaned with 75% alcohol to eliminate olfactory cues.

### Fear Conditioning Test

To measure learning and memory abilities (Wu et al., 2015b), we employed the fear conditioning paradigm (30 cm long × 26 cm wide × 22 cm high, XR-XC404, Shanghai Softmaze Information Technology Co., Ltd.). Each mouse was exposed to the conditioning chamber for 3 min of accommodation; one tone/foot shock pairing (tone, 30 s, 65 dB, 3 kHz; foot-shock, 3 s, 0.75 mA) was then delivered. The contextual fear conditioning test was performed 24 h later by placing each mouse back in the same test chamber for 5 min without any stimulation. Two hours later, each mouse was placed in a novel chamber that differed in shape, color, and smell, and the same tone was presented for 3 min without the foot shock to evaluate tone fear conditioning. Cognitive deficits in the test were assessed by measuring the length of “freezing behavior,” which is defined as a completely immobile posture except for respiratory efforts. Freezing behaviors were automatically recorded by the video tracking system.

### Morris Water Maze (MWM) Tests

One week after gas inhalation, the mice (*n* = 12 for each group) were subjected to MWM (XR-XM101; Shanghai Softmaze Information Technology Co., Ltd.) to test their spatial learning and memory as previously described (Wu et al., 2017). Briefly, this consisted of two phases, a training phase for five consecutive days and a probe trial phase on day 6. In the training phase, each mouse was allowed to face the pool wall in four random places (N, S, E, and W) in the pool to find the fixed platform. Release positions were randomly predetermined. The trial was terminated once the mouse reached the platform. If the mouse failed to reach the platform within 60 s, it would be guided to the platform and allowed to stay for 15 s, and the latency was then recorded for 60 s. In the probe test, a single-probe trial was conducted with the original platform removed 24 h after the last training session. Each mouse was allowed to swim for 60 s to test their memory (platform-crossing times and target quadrant traveling time).

### Statistical Analysis

Data are presented as the mean ± SEM and were analyzed by the Statistical Product for Social Sciences (SPSS; version 18.0, IL, United States). The difference between the groups was determined by one-way analysis of variance followed by the Bonferroni test. Comparisons for the spatial training sessions of MWM were performed by repeated two-way ANOVA followed by the LSD test. A *p*-value <0.05 was considered statistically significant.

## Results

### MCC950 Inhibits the Activation of the NLRP3 Inflammasome in the Hippocampus of Aged Mice Exposed to Isoflurane

**FIGURE 1:**
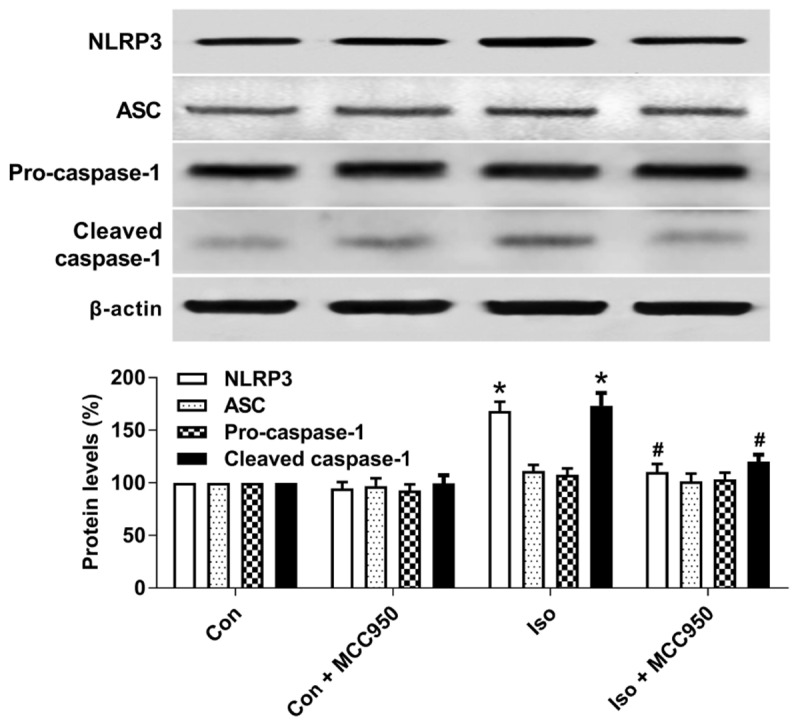
MCC950 pretreatment inhibited the activation of the NLRP3 inflammasome in the hippocampus of aged mice exposed to isoflurane. MCC950 (10 mg/kg) or PBS was intraperitoneally administered to the mice with a volume of 0.4 mL/kg 30 min before gas inhalation. Anesthesia was induced by placing the mice in an anesthetizing chamber prefilled with 1.8% isoflurane plus 30% O_2_ for 10 min and then changed to 1.5% isoflurane for 110 min. For control experiments, 30% O_2_ was delivered for 2 h at an identical flow rate. Con, mice treated with PBS and 30% O_2_; Con + MCC950, mice treated with MCC950 and 30% O_2_; Iso, mice treated with PBS and isoflurane; Iso + MCC950, mice treated with MCC950 and isoflurane. The definition of Con/Con + MCC950/Iso/Iso + MCC950 is the same as in the following figures. The protein levels of NLRP3, ASC, Pro-caspase-1, and cleaved caspase-1 were determined with the fresh homogenates of hippocampal tissues obtained from the aged mice (see section “Materials and Methods”) 24 h after gas inhalation. Values are presented as mean ± SEM (*n* = 6 mice/group). ^∗^*p* < 0.05 versus the control group; ^#^*p* < 0.05 versus the isoflurane group.

**FIGURE 2:**
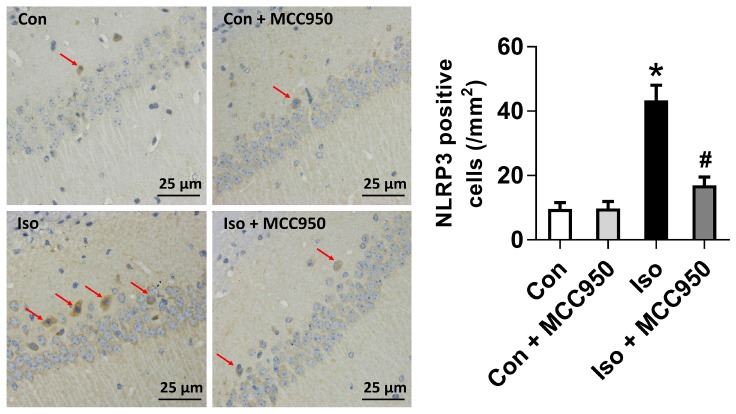
The number of NLRP3-positive cells was diminished by MCC950 pretreatment in the hippocampal CA1 regions. Representative images of NLRP3 immunohistochemical (IHC) staining in the hippocampal CA1 region are shown. Cells with brownish-yellow cytoplasm are positive for NLRP3. Scale bar = 25 μm. Right panel presents statistical data from the four experimental groups. Values are presented as mean ± SEM (*n* = 6 mice/group). ^∗^*p* < 0.05 versus the control group; ^#^*p* < 0.05 versus the isoflurane group.

The NLRP3 inflammasome, a component of the inflammatory process, is highly expressed in various inflammatory diseases (Wu et al., 2015b; Mangan et al., 2018). MCC950 is the most specific and well characterized NLRP3 inhibitor to date and has been tested in a diverse array of NLRP3-involved inflammatory diseases (Mangan et al., 2018; Shen et al., 2018). We previously showed that isoflurane induced NLRP3 inflammasome activation in the hippocampus of aged mice (Wu et al., 2015a). Here, we examined whether the inflammasome activation is inhibited by MCC950 treatment. Our results showed that the protein levels of NLRP3 and cleaved caspase-1 (Figure 1) and the number of NLRP3-positive cells (Figure 2) were increased 24 h after 2 h isoflurane exposure. Notably, the increases were reversed by the administration of MCC950 (Figures 1, 2). The adaptor protein ASC and pro-caspase-1 showed no differences among the four groups (Figure 1). Our results indicate that isoflurane-induced NLRP3 inflammasome activation is successfully inhibited by MCC950 administration.

### Inhibiting the NLRP3 Inflammasome With MCC950 Ameliorates Pyroptosis in the Hippocampus of Aged Mice Exposed to Isoflurane

**FIGURE 3:**
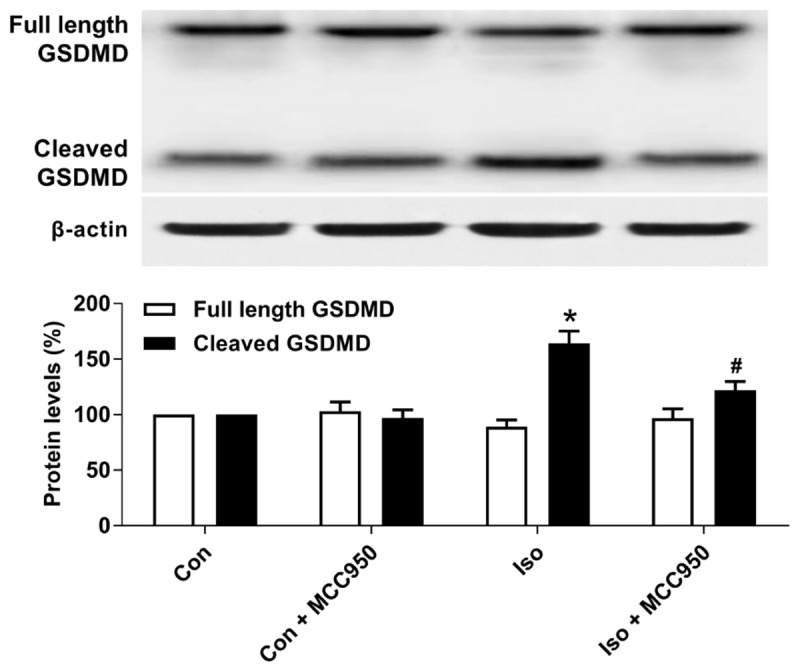
Effects of isoflurane and MCC950 pretreatment on pyroptosis in the hippocampus of aged mice. Representative Western blotting and quantitative analysis of protein levels of full length GSDMD and cleaved GSDMD in the hippocampal tissues. Values are presented as mean ± SEM (*n* = 6 mice/group). ^∗^*p* < 0.05 versus the control group; ^#^*p* < 0.05 versus the isoflurane group.

**FIGURE 4:**
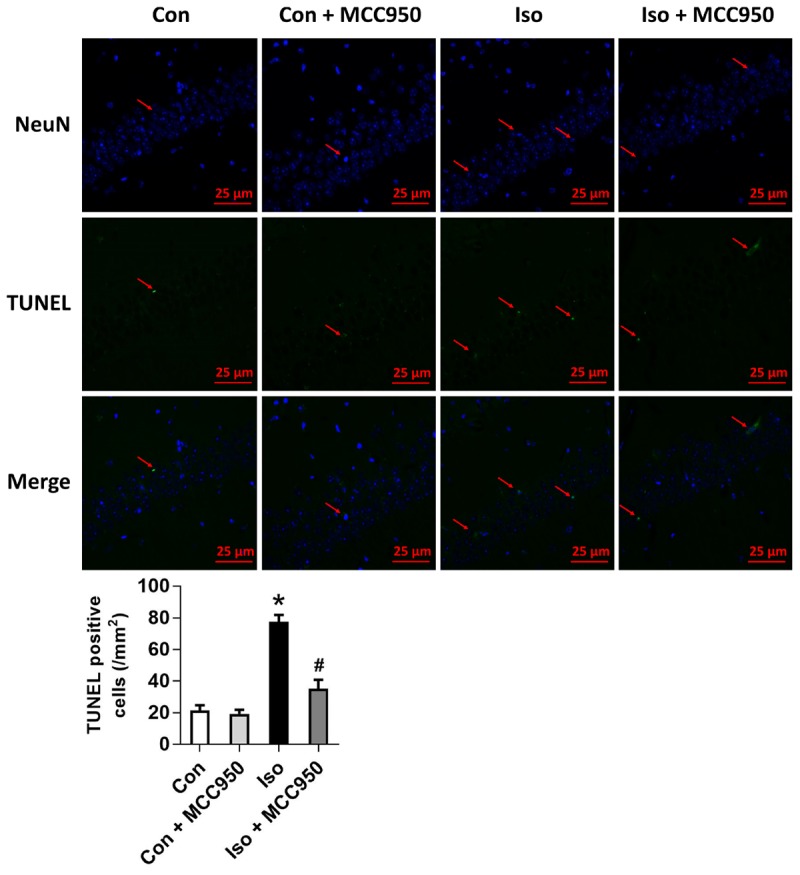
Effects of isoflurane and MCC950 pretreatment on the number of TUNEL-positive cells in the hippocampal CA1 region. Representative images of TUNEL staining in the hippocampal CA1 region are shown. Green color indicates TUNEL-positive cells and blue indicates NeuN-stained nucleus. Scale bar = 25 μm. The lower panel shows statistical numbers of TUNEL-positive cells from the four experimental groups. Values are presented as mean ± SEM (*n* = 6 mice/group). ^∗^*p* < 0.05 versus the control group; ^#^*p* < 0.05 versus the isoflurane group.

Pyroptosis was recently characterized to be NLRP3 inflammasome-dependent. NLRP3 inflammasome activation governs the cleavage of caspase-1, resulting in the maturation of effector pro-inflammatory cytokines and mediating pyroptosis (He et al., 2016a; Dempsey et al., 2017; Jiang et al., 2018). Recent progress has identified the protein GSDMD as the substrate of active caspase-1 and caspase-11 and the executor of proptosis (Kayagaki et al., 2015; Shi et al., 2015). Active caspase-1 (canonical inflammasome pathway) or caspase-11 (non-canonical inflammasome pathway) enzymatically cleaves GSDMD into two fragments (the N domain and C domain) (Shi et al., 2015, 2017; Gaidt and Hornung, 2016; Sborgi et al., 2016; Aglietti and Dueber, 2017). Furthermore, the GSDMD-N domain forms pores on lipid membranes and induces pyroptosis through cell membrane disruption. Here, we showed that isoflurane GA induced an increase in cleaved GSDMD but not full length GSDMD in the hippocampus of aged mice (Figure 3), implying that pyroptosis was activated during isoflurane GA. In addition, the TUNEL assay showed that the percentage of dead neurons in the isoflurane group was higher than that in the control group (Figure 4). Interestingly, MCC950 pretreatment attenuated the pyroptotic cell death induced by isoflurane GA (Figures 3, 4). These results suggest that inhibiting the NLRP3 inflammasome with MCC950 ameliorates pyroptosis in the hippocampus of aged mice exposed to isoflurane.

### MCC950 Suppresses the Release of Inflammatory Cytokines in the Hippocampus of Aged Mice Exposed to Isoflurane

**FIGURE 5:**
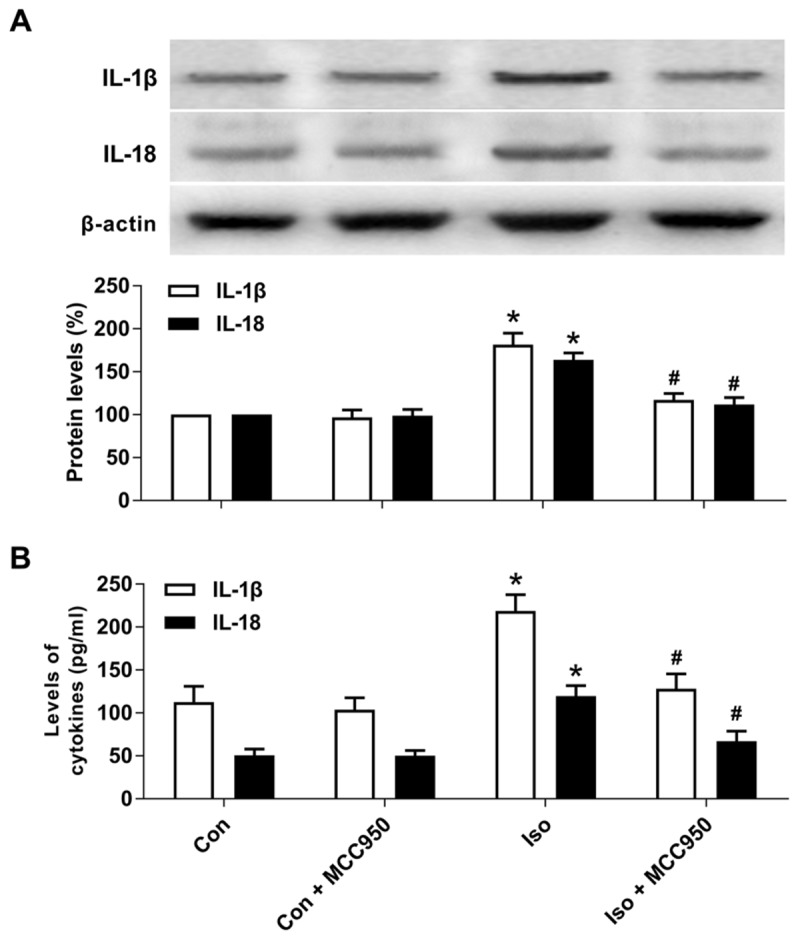
MCC950 pretreatment suppressed the release of inflammatory cytokines in the hippocampus of aged mice exposed to isoflurane. **(A)** Western blotting analyses of IL-1β and IL-18 levels. **(B)** ELISA assays of IL-1β and IL-18 levels. Values are presented as mean ± SEM (*n* = 6 mice/group). ^∗^*p* < 0.05 versus the control group; ^#^*p* < 0.05 versus the isoflurane group.

Pyroptotic cell death involves the formation of a plasma membrane pore, cell swelling, and rupture of the plasma membrane, which results in the release of abundant inflammatory factors (Shi et al., 2015, 2017; Aglietti and Dueber, 2017). Our results showed that the inflammatory cytokines IL-1β and IL-18 (Figure 5) were increased 24 h after isoflurane exposure in the hippocampus of the aged mice, while MCC950 pretreatment significantly suppressed the secretion of IL-1β and IL-18 (Figure 5). Therefore, our results suggest that inhibiting NLRP3 inflammasome-dependent pyroptosis with MCC950 suppresses the secretion of inflammatory cytokines in the hippocampus of aged mice exposed to isoflurane.

### MCC950 Reverses Neuronal Morphological Damage in the Hippocampus of Aged Mice Exposed to Isoflurane

**FIGURE 6:**
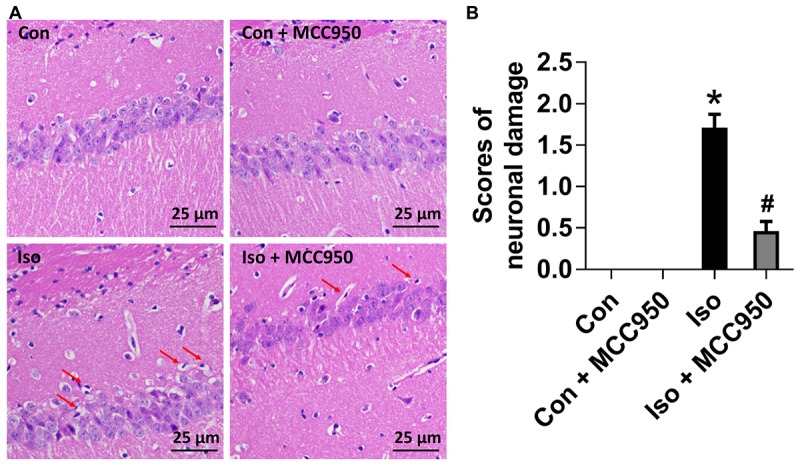
MCC950 pretreatment reversed isoflurane-induced neuronal morphological damage in the hippocampus of aged mice. **(A)** Representative pictures of HE staining in the hippocampal CA1 region are shown. Red arrows: Cells arranged in a disorderly manner, cytoplasm and nucleolus deeply dyed with nuclear pyknosis. Scale bar = 25 μm. **(B)** The right panel shows statistical scores of neuronal damage. Values are presented as mean ± SEM (*n* = 6 mice/group). ^∗^*p* < 0.05 versus the control group; ^#^*p* < 0.05 versus the isoflurane group.

To evaluate the neuroprotective effects of MCC950 on isoflurane-induced neuronal morphological damage, we conducted HE staining on brain sections of the hippocampal CA1 region 24 h after gas inhalation. The mice in the isoflurane group showed the most condensed cell bodies, as well as most extensive karyopyknosis and karyolysis (Figure 6). In contrast, the mice with MCC950 pretreatment showed significant decreases in abnormal neurons and neuronal damage scores (Figure 6). This result suggests that MCC950 protects hippocampal neurons from isoflurane-induced morphological damage.

### MCC950 Attenuates Isoflurane-Induced Cognitive Impairment in Aged Mice

**FIGURE 7:**
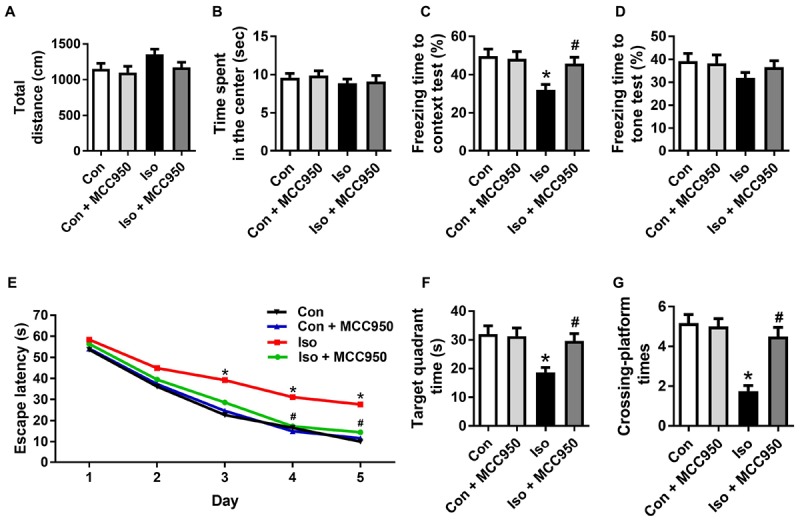
MCC950 pretreatment prevented isoflurane-induced cognitive impairment in aged mice. **(A)** Total distance traveled and **(B)** time spent in the center in the open field test. **(C)** Freezing time to context and **(D)** freezing time to tone in the fear conditioning test. **(E)** Escape latency for every day during the spatial training of MWM. **(F)** Time spent in the target quadrant and **(G)** crossing-platform times in the probe trial of MWM. Values are presented as mean ± SEM (*n* = 12 mice/group). ^∗^*p* < 0.05 versus the control group; ^#^*p* < 0.05 versus the isoflurane group. The procedures of the behavioral tests are in the section “Materials and Methods.” MWM, Morris water maze.

To further evaluate the neuroprotective effects of MCC950 on isoflurane-induced cognitive impairment, the open field, fear conditioning and MWM tests were performed. The open field test showed no difference among the four groups in locomotor activity as reflected by the total distance (Figure 7A) and time spent in the center (Figure 7B), which excluded the possibility that locomotor activity *per se* affected the results in the fear conditioning and MWM tests.

The fear conditioning test showed a decreased percentage of freezing time in the 24 h context test, which reflected impairment of hippocampus-dependent memory in isoflurane-treated mice compared to that in the control mice (Figure 7C). The MWM tests showed a long escape latency in the training test (Figure 7E) as well as decreased target quadrant time (Figure 7F) and crossing-platform times (Figure 7G) in the probe trial in the isoflurane-treated mice. Interestingly, all of the changes in the behavioral tests (Figure 7) were counteracted by MCC950 pretreatment. These results indicate that isoflurane GA for 2 h led to cognitive impairment in the aged mice and that MCC950 (10 mg/kg) intraperitoneal treatment has a therapeutic effect on isoflurane-induced cognitive impairment.

## Discussion

We previously demonstrated that isoflurane GA induced hippocampal NLRP3 inflammasome activation and cognitive deficits in aged mice (Wu et al., 2015a). In the present study, we showed that the isoflurane-induced activation of the NLRP3 inflammasome triggered neuronal damage in a pyroptotic manner. Pretreatment with NLRP3 inflammasome inhibitor MCC950 significantly suppressed the expressions of NLRP3 inflammasome and cleaved caspase-1 and the associated release of the inflammatory cytokines IL-1β and IL-18 in the hippocampus of aged mice. Moreover, MCC950 protected aged mice from isoflurane-induced neuronal damage and cognitive impairment. Our results collectively suggest that NLRP3 inflammasome-dependent pyroptosis plays a negative role in the pathogenesis of isoflurane-induced cognitive impairment in aged mice and that MCC950 acts as a potential therapeutic compound for the treatment of POCD.

The NLRP3 inflammasome is a critical component of inflammasomes that functions through an interaction with apoptosis-associated speck-like protein (ASC) and the subsequent recruitment of the precursor form of caspase-1, leading to the cleavage of caspase-1 and the maturation and secretion of IL-1β and IL-18 (He et al., 2016a; Dempsey et al., 2017; Jiang et al., 2018). Consistent with previous studies, we found that isoflurane induced the upregulation of NLRP3, the cleavage of caspase-1, and the secretion of IL-1β and IL-18 in the hippocampus of aged mice. Unexpectedly, isoflurane did not increase the expression of ASC in the present study. It is possible that other proteins are involved in the formation of the NLRP3 inflammasome, such as NIMA-related kinases (He et al., 2016b) and protein kinase D (Zhang et al., 2017). Moreover, activated NLRP3 inflammasome-caspase-1 can cleave GSDMD into the GSDMD-N domain, which further forms pores on lipid membranes and induces pyroptosis (Shi et al., 2015, 2017; Gaidt and Hornung, 2016; Sborgi et al., 2016; Aglietti and Dueber, 2017). Thus, our study also showed that cleaved GSDMD, but not full length GSDMD, was upregulated in the hippocampus of aged mice exposed to isoflurane. Our data provide preliminary evidence that pyroptosis is likely involved in GA-induced neuroinflammation.

Activation of the NLRP3 inflammasome and pyroptosis may cause neuronal damage, cognitive dysfunction, and even death (Bordon, 2012; Tan et al., 2014; Ding et al., 2018). The hippocampus is known to be critical for contextual and spatial memory, while acute pathological conditions, such as ischemia, hypoglycemia and other neurological conditions, can cause damage to the hippocampus (Squire, 1992; Bartsch and Wulff, 2015; Wu et al., 2015b). This acute damage results in the selective cell death of hippocampal CA1 neurons, whereas the dentate gyrus (DG), CA3, and most cortical neurons appear to be more resistant (Bartsch and Wulff, 2015; Bartsch et al., 2015). Thus, we examined neuronal damage in the hippocampal CA1 region in the present study and found that mice in the isoflurane group exhibited massively injured neurons in this region. Moreover, the mice in the isoflurane group performed significantly worse in the contextual fear conditioning test and MWM tests than those in the control group, showing that hippocampus-dependent contextual memory and spatial learning and memory were impaired. Altogether, previous studies as well as our study suggest that NLRP3 inflammasome-dependent pyroptosis is a key regulator of the underlying mechanisms of isoflurane-induced neuronal damage and cognitive impairment in aged mice.

MCC950 is a newly developed, selective, small-molecule NLRP3 inflammasome inhibitor that can freely cross the blood–brain barrier (Chen et al., 2017; Qi et al., 2018). Zhai et al. (2018) recently reported that the intraperitoneal administration of MCC950 (10 mg/kg) could downregulate the hippocampal NLRP3, ASC, and IL-1β expression levels and ameliorate cognitive impairment in diabetic db/db mice. In line with these data, our study showed that MCC950 (10 mg/kg) intraperitoneal treatment was efficacious in ameliorating cognitive impairment in aged mice exposed to isoflurane. In addition, histochemical and biochemical findings supported that MCC950 protected aged mice against hippocampal NLRP3 inflammasome activation, inflammatory cytokines secretion, pyroptotic cell death, and neuronal morphological damage. These findings provided evidence that the administration of the specific NLRP3 inhibitor MCC950 ameliorates the pyroptosis and cognitive impairment caused by isoflurane anesthesia in the aged mice.

## Conclusion

This is the first study to our knowledge to show that pyroptosis is involved in isoflurane-induced cognitive impairment in aged mice and that inhibiting the NLRP3 inflammasome with MCC950 ameliorates the neuroinflammation, pyroptosis, neuronal damage, and cognitive impairment caused by isoflurane anesthesia in these mice. This work suggests a new strategy for reversing age- and/or neuroinflammation-related impairment in GA-induced neurotoxicity and cognitive deficits, supporting the notion that MCC950 may have therapeutic benefits for elderly patients with POCD.

## Author Contributions

YF and LD performed the experiments and acquired the data. YF, LD, QF, ZZ, and JZ analyzed and interpreted the data. JW conceived and designed the experiments. YF and JW wrote the manuscript. YF, LD, QF, ZZ, JZ, and GL contributed to the acquisition and analysis of data. All authors read and approved the final manuscript.

## Conflict of Interest Statement

The authors declare that the research was conducted in the absence of any commercial or financial relationships that could be construed as a potential conflict of interest.
